# Supplementary material for: Tracking the introduction and spread of SARS-CoV-2 in coastal Kenya
Source: Nat Commun. 2021 Aug 10;12:4809. doi: 10.1038/s41467-021-25137-x (PMC8355311; doi:10.1038/s41467-021-25137-x)
Supplement: Supplementary file 1 — Supplementary Information [file 41467_2021_25137_MOESM1_ESM.pdf]

TITLE: Tracking the introduction and spread of SARS-CoV-2 in coastal Kenya

George Githinji[1,2*], Zaydah R. de Laurent[1], Khadija Said Mohammed[1], Donwilliams O. Omuoyo[1], Peter M. Macharia[3], John M. Morobe[1], Edward Otieno[1], Samson M. Kinyanjui[1,4], Ambrose Agweyu[1], Eric Maitha[5], Ben Kitole[5], Thani Suleiman[6], Mohamed Mwakinangu[7], John Nyambu[8], John Otieno[9], Barke Salim[10], Kadondi Kasera[11], John Kiiru[11], Rashid Aman[11], Edwine Barasa[4,12], George Warimwe[1,4], Philip Bejon[1,4], Benjamin Tsofa[1], Lynette Isabella Ochola-Oyier[1], D. James Nokes[1,13], Charles N. Agoti[1,14]

Supplementary figures

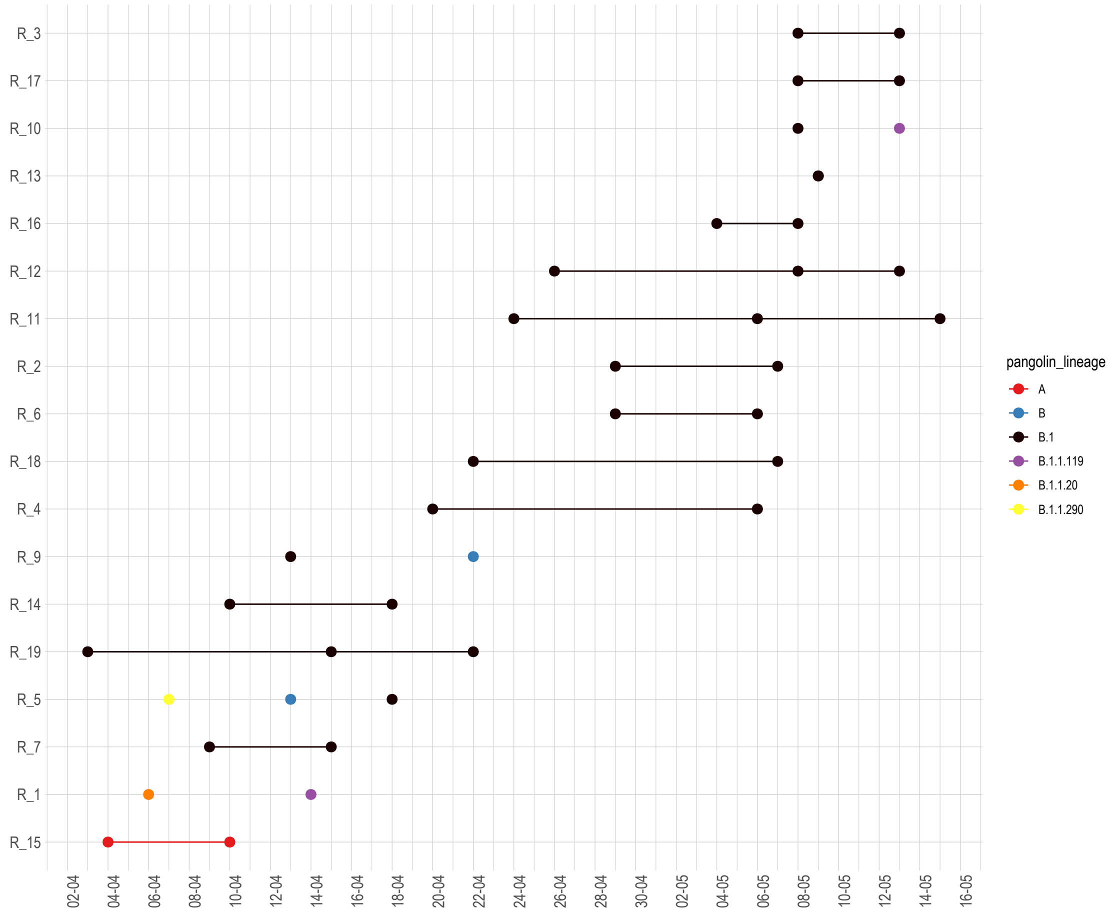

Supplementary figure 1: A plot showing the lineage concordance among samples repeatedly taken from the same patient. The horizontal axis shows the day of sample collection, and the vertical axis shows the individual. The shaded circles represent the PANGO lineage.

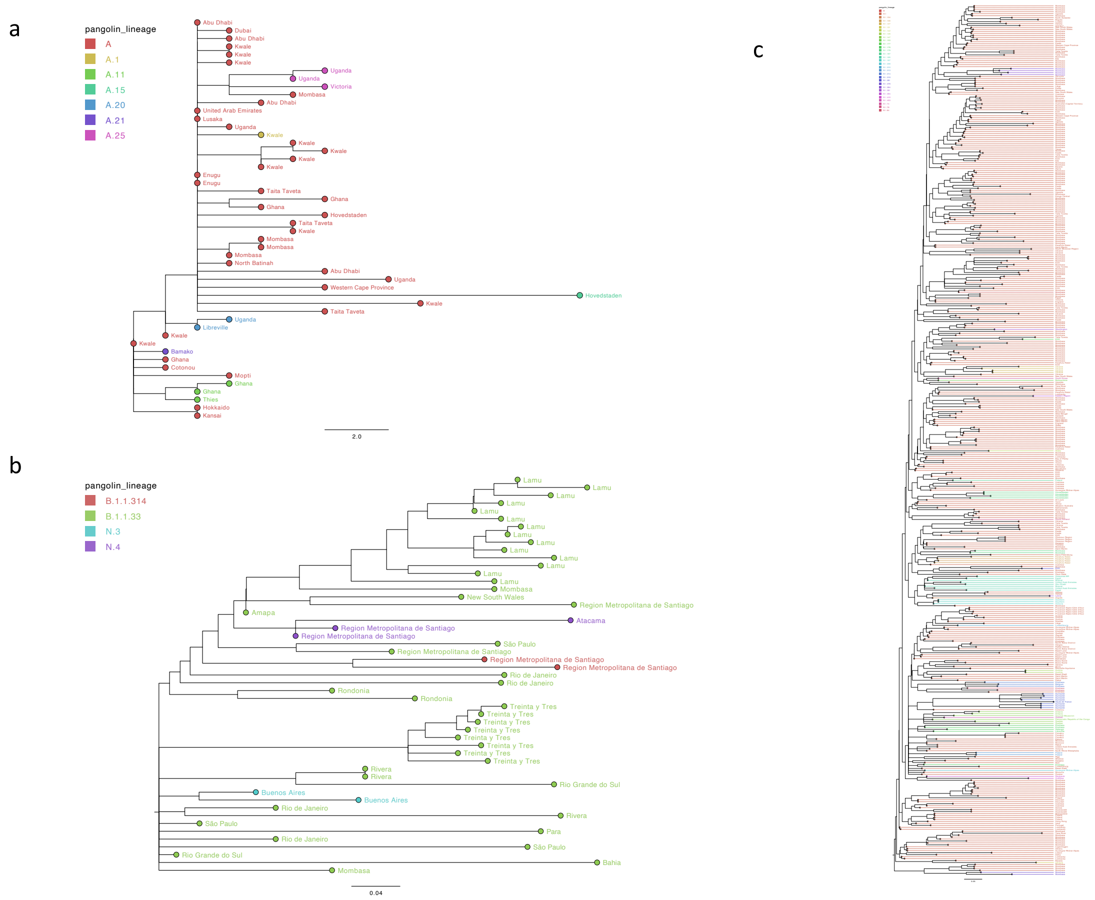

Supplementary figure 2: **a.** A maximum likelihood phylogenetic tree showing lineage A sequences from coastal Kenya and in context with global sequences. The nodes are coloured based on the lineage the tip labels represent the region from which the samples were collected. **b.** A maximum likelihood phylogenetic tree showing sequences consistent with lineage B.1.1.33. the horizontal axis represents time divergence, and the nodes and tip colours represent the lineage. **c.** A phylogenetic tree of B.1 lineage of the Kenyan sequences showing the lineage assignment in the context of global sequences. Lineage B.1 was the most extensive lineage. The branches are coloured based on the inferred geographic location of the internal nodes.

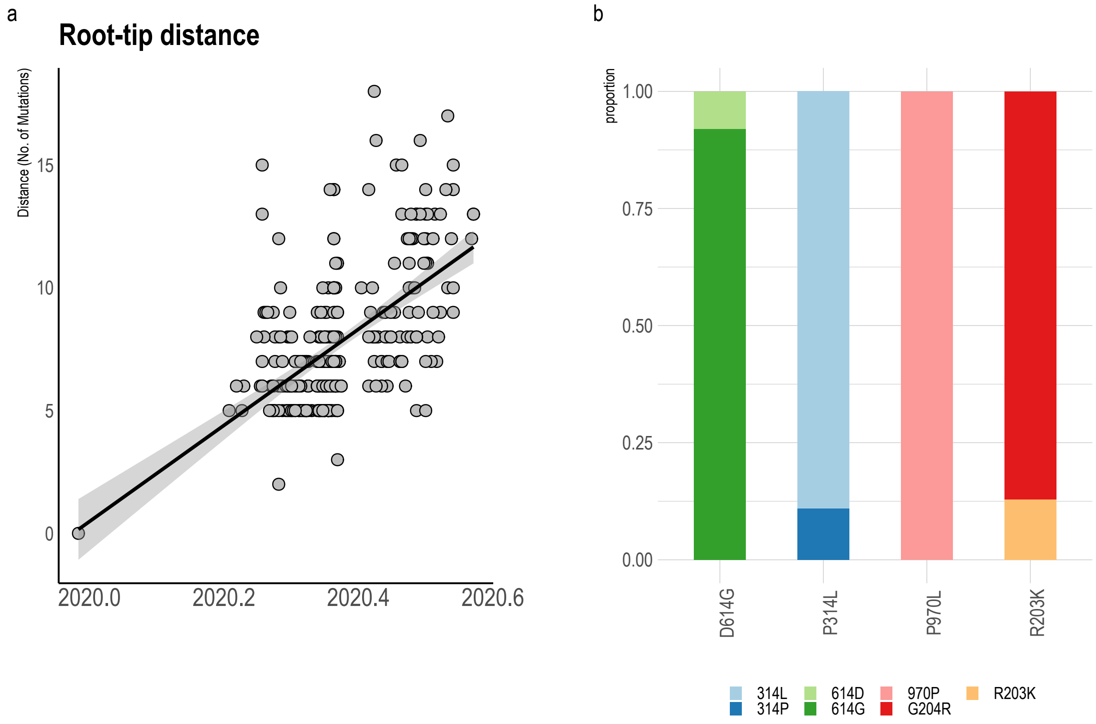

Supplementary figure 3: a. **A summary of the sequence divergence among sequences from the coast. a** A root to tip divergence plot showing the overall number of mutations among 311 SARS-CoV-2 collected from the coast against time (x-axis) obtained using Tempest to obtain the phylogenetic signal. Each point represents the distance to the root for each of the sequences used in the phylogenetic analysis. The grey shaded area represents the 95% confidence interval. The overall rate estimate for these sequences was 17.695 substitutions per year. **b** The relative proportion (y-axis) of mutations of interest (x-axis) among the early sequences collected from the Kenyan coast.

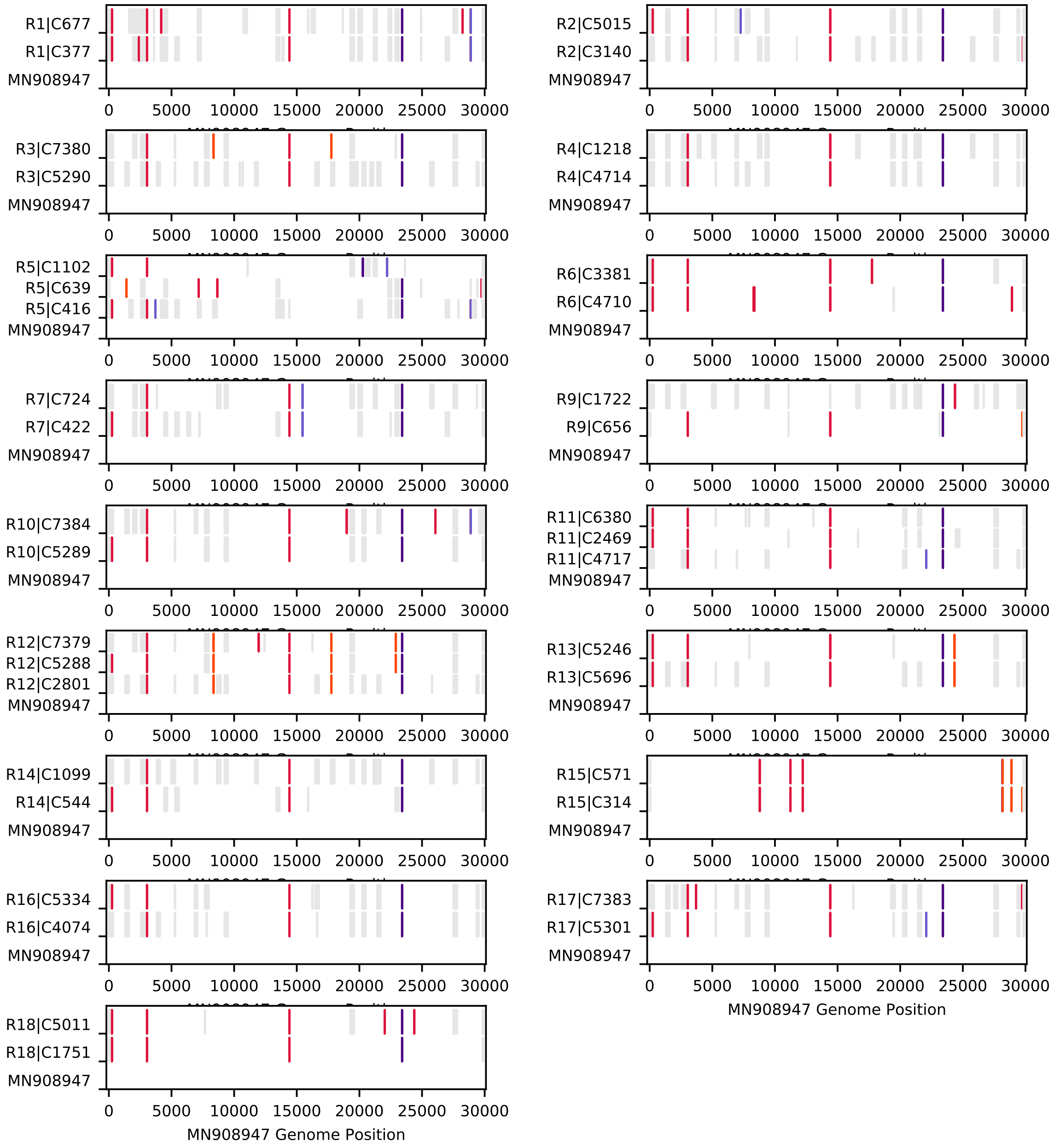

Supplementary figure 4: Highliter plots of nucleotide changes among sequences collected repeatedly from 17 individuals. In each panel, the x-axis represents the genome position. Grey regions represent regions with missing information and would be represented by string on N. The reference genome is shown at the bottom of each panel.

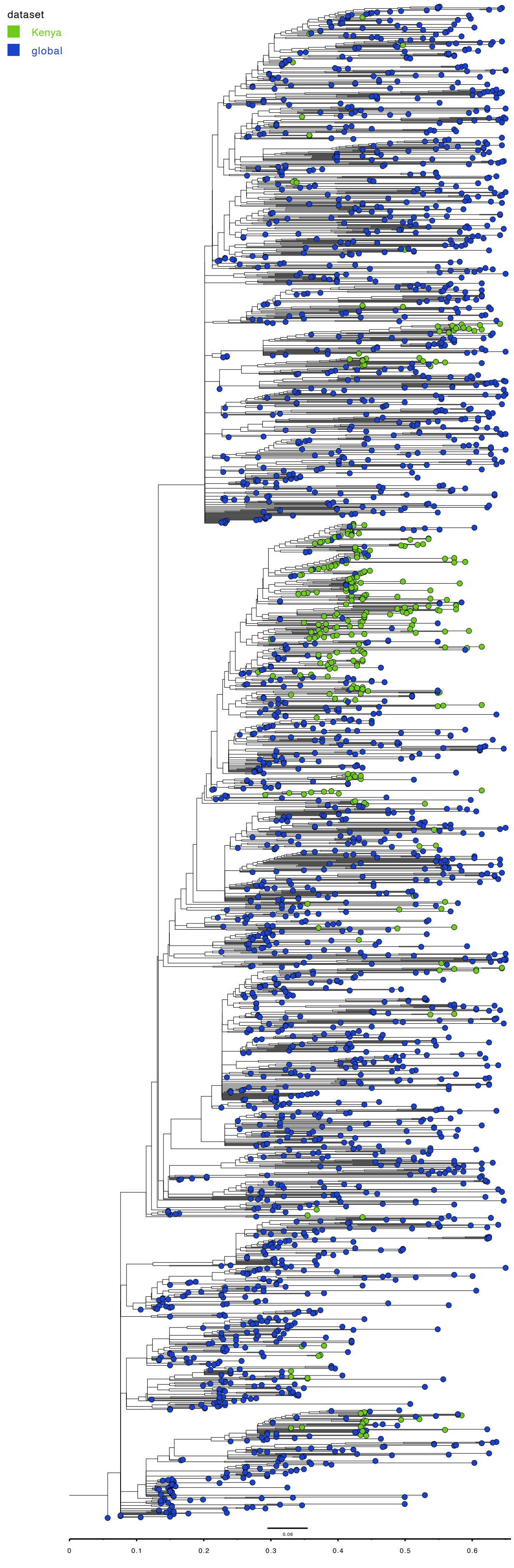
dataset
Kenya
global
0.06
0
0.1
0.2
0.3
0.4
0.5
0.6

Supplementary figure 5: A time resolved phylogenetic tree with branch length in units of years showing the evolutionary relationship between 311 SARS-CoV-2 sequenced samples collected from coastal Kenya in relation to 2,078 global sequences. The green tips represent sequences collected from coastal Kenya and blue tips represent sequences collected from elsewhere in the globe.

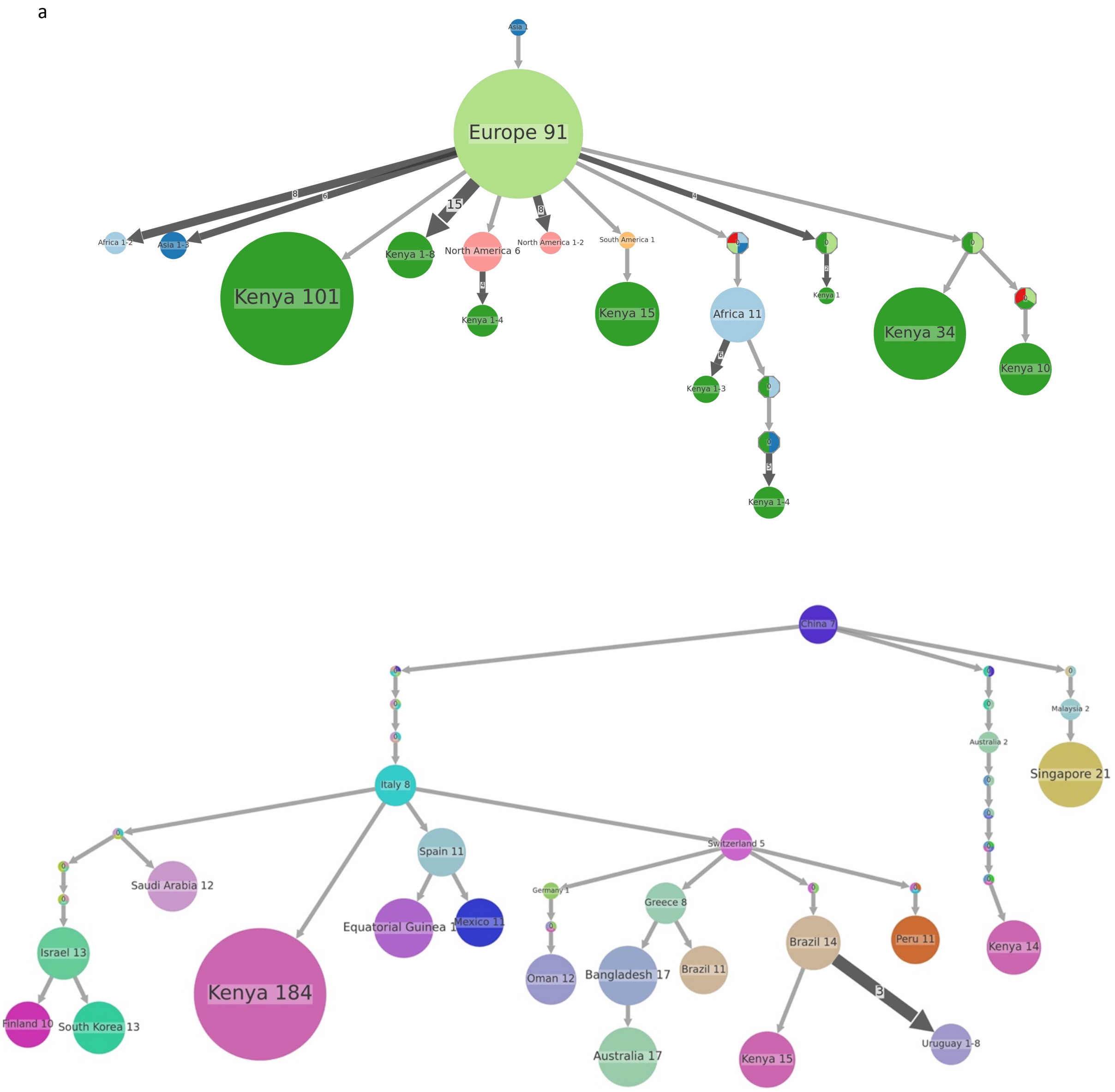

Supplementary figure 6: A summary of ancestral reconstruction and estimation of the number of introductions using pastML. **a** A summary of the number of estimated introductions based on ancestral reconstruction with PastML (MPPA+F81) with geolocations aggregated by continental regions. The nodes are aggregated by broad geographic regions and denoted by the colours. The edges denoted transmission events. **b** A summary of estimated introductions based on ancestral

reconstruction using PastML (MPPA+F81) with geolocations aggregated by country. Internal nodes are collapsed based on shared reconstructed ancestry and the edges denote putative transmission events.

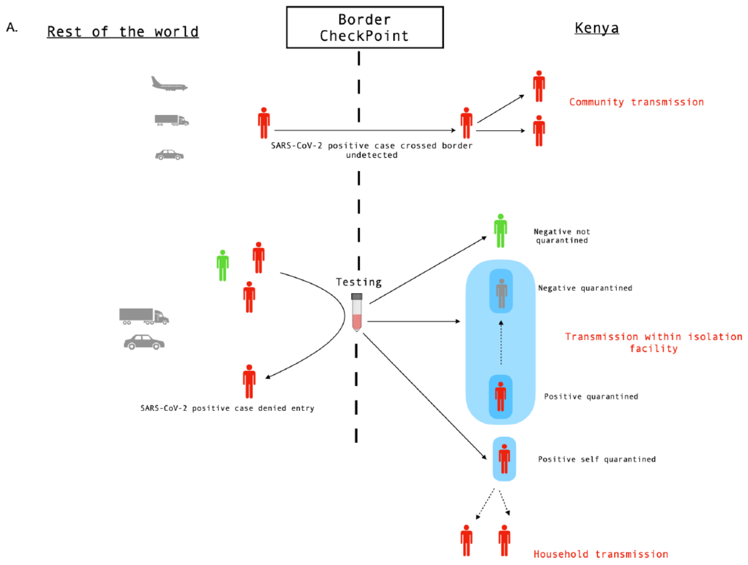

Supplementary figure 7: An importation model for SARS-CoV-2 cases. Cases are likely to have been imported into the coast region undetected through international ports of entry, travellers and commercial truck drivers from Nairobi and neighbouring countries despite screening of commercial truck drivers and turning back individuals who tested positive at the points of entry with Tanzania. Multiple undetected cases are likely to have been the source of the major epidemic that occurred in Mombasa

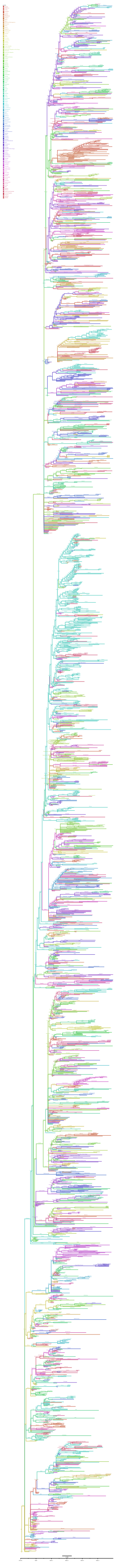

Supplementary figure 8: A phylogenetic tree showing the global context of the Kenyan sequences coloured by ancestral state reconstruction. (A high resolution image is available at https://github.com/george-githinji/sars-cov-2-early-phase-manuscript/blob/main/figures/supplementary%20figure%208.pdf)

Supplementary tables

	Kilifi (N=21)	Kwale (N=40)	Lamu (N=18)	Mombasa (N=287)	Taita Taveta (N=36)	Tana River (N=4)	Total (N=406)
**Sex**							
Female	9 (42.9%)	2 (5.0%)	9 (50.0%)	89 (31.0%)	0 (0%)	1 (25.0%)	110 (27.1%)
Male	10 (47.6%)	36 (90.0%)	9 (50.0%)	176 (61.3%)	34 (94.4%)	3 (75.0%)	268 (66.0%)
Unknown	2 (9.5%)	2 (5.0%)	0 (0%)	22 (7.7%)	2 (5.6%)	0 (0%)	28 (6.9%)
**Age**							
Mean (SD)	33.5 (9.69)	35.6 (9.81)	41.4 (17.3)	40.7 (16.2)	39.3 (9.62)	46.0 (22.6)	39.8 (15.0)
Median [Min, Max]	34.0 [12.0, 63.0]	33.0 [19.0, 56.0]	39.0 [21.0, 70.0]	40.0 [1.00, 85.0]	40.0 [22.0, 56.0]	40.5 [28.0, 75.0]	39.0 [1.00, 85.0]
Missing	0 (0%)	1 (2.5%)	1 (5.6%)	14 (4.9%)	0 (0%)	0 (0%)	16 (3.9%)
**Age category**							
0-9	0 (0%)	0 (0%)	0 (0%)	10 (3.5%)	0 (0%)	0 (0%)	10 (2.5%)
10-19	1 (4.8%)	1 (2.5%)	0 (0%)	10 (3.5%)	0 (0%)	0 (0%)	12 (3.0%)
20-29	3 (14.3%)	10 (25.0%)	4 (22.2%)	41 (14.3%)	7 (19.4%)	2 (50.0%)	67 (16.5%)
30-39	13 (61.9%)	15 (37.5%)	5 (27.8%)	71 (24.7%)	10 (27.8%)	0 (0%)	114 (28.1%)
40-49	3 (14.3%)	8 (20.0%)	4 (22.2%)	55 (19.2%)	12 (33.3%)	0 (0%)	82 (20.2%)
50-59	0 (0%)	5 (12.5%)	0 (0%)	53 (18.5%)	7 (19.4%)	1 (25.0%)	66 (16.3%)
60-69	1 (4.8%)	0 (0%)	1 (5.6%)	23 (8.0%)	0 (0%)	0 (0%)	25 (6.2%)
70-79	0 (0%)	0 (0%)	3 (16.7%)	6 (2.1%)	0 (0%)	1 (25.0%)	10 (2.5%)
80-89	0 (0%)	0 (0%)	0 (0%)	4 (1.4%)	0 (0%)	0 (0%)	4 (1.0%)
Missing	0 (0%)	1 (2.5%)	1 (5.6%)	14 (4.9%)	0 (0%)	0 (0%)	16 (3.9%)

	Kilifi (N=21)	Kwale (N=40)	Lamu (N=18)	Mombasa (N=287)	Taita Taveta (N=36)	Tana River (N=4)	Total (N=406)
**Travel information**							
Border	1 (4.8%)	15 (37.5%)	0 (0%)	19 (6.6%)	32 (88.9%)	0 (0%)	67 (16.5%)
Local	9 (42.9%)	1 (2.5%)	15 (83.3%)	141 (49.1%)	0 (0%)	2 (50.0%)	168 (41.4%)
Travel associated	3 (14.3%)	23 (57.5%)	2 (11.1%)	13 (4.5%)	1 (2.8%)	0 (0%)	42 (10.3%)
Unknown	8 (38.1%)	1 (2.5%)	1 (5.6%)	114 (39.7%)	3 (8.3%)	2 (50.0%)	129 (31.8%)
**Symptoms**							
Asymptomatic	15 (71.4%)	22 (55.0%)	11 (61.1%)	155 (54.0%)	27 (75.0%)	1 (25.0%)	231 (56.9%)
Symptomatic	3 (14.3%)	0 (0%)	3 (16.7%)	43 (15.0%)	1 (2.8%)	0 (0%)	50 (12.3%)
Unknown	3 (14.3%)	18 (45.0%)	4 (22.2%)	89 (31.0%)	8 (22.2%)	3 (75.0%)	125 (30.8%)

Supplementary Table 1: Demographic characteristics of SARS-CoV-2 samples collected between March and July 2020 (n=406) from coastal Kenya stratified by county. The case history demographic characteristic was derived from both self-reported travel history and presentation at a border point. Local case-history refers to individuals that did not report a history of travel and neither were they screened at a port of entry. Individuals whose case histories were not filled, or information was missing were labelled as unknown.

Phase	Eligibility	Comments
Phase 1	• Showed specific symptoms of respiratory illness<br>• Recent international travel<br>• Close contacts of a confirmed case	Before confirmed case<br>(December 2019 – 11th March 2020)
Phase 2	• Showed specific symptoms of respiratory illness<br>• Recent international travel<br>• Close contacts of a confirmed case<br>• International visitors were taken to isolation centres	After first confirmed case<br>(12th March 2020 – 5th May 2020)
Phase 3	• Target testing was rolled out at Kenya Ports Authority and the general public in Mombasa	After suspected community transmission<br>(6th May 2020)
Phase 4	• Targeted testing of truck drivers entering Kenya	This was partly a response to increased reporting of cases by Uganda border surveillance team

Supplementary Table 2: A table summarising four large-scale public health responses that were rolled out by the Kenyan Ministry of Health (MoH) in response to increased number of SARS-CoV-2 PCR positive cases between March and July 2020. The eligibility criteria influenced the decision on who was PCR tested and thereby the number of confirmed cases.

	Border (N=8)	Local (N=10)	Travelled (N=6)	Unknown (N=9)
**Lineage**				
A	2	-	-	-
B	1	-	-	1
B.1.1.1	1	-	2	-
B.1.387	1	-	-	-
B.4	3	-	-	-
B.1.1.119	-	1	-	-
B.1.1.254	-	1	-	-
B.1.1.33	-	1	-	-
B.1.1.70	-	1	-	-
B.1.126	-	1	-	-
B.1.179	-	1	-	-
B.1.281	-	1	-	-
B.1.378	-	1	-	-
B.1.416	-	1	-	-
N.4	-	1	-	-
A.1	-	-	1	-
B.1	-	-	1	-
B.1.1.111	-	-	1	-
B.1.1.290	-	-	1	-
B.1.1.20	-	-	-	1

	Border (N=8)	Local (N=10)	Travelled (N=6)	Unknown (N=9)
B.1.1.291	-	-	-	1
B.1.1.300	-	-	-	1
B.1.177	-	-	-	1
B.1.222	-	-	-	1
B.1.384	-	-	-	1
B.1.399	-	-	-	1
B.29	-	-	-	1

Supplementary Table 3: A summary of initial cases for each lineage that were observed among the early SARS-CoV-2 coastal Kenya sequences collected between March and 31st July 2020 stratified by the potential entry source. Border means that the sample was identified or collected at a point of entry (PoE) while travelled refers to cases where individuals reported a history of travel in the last 14 days. Unknown refers to cases where travel information was not captured at the time of sample collection.

	Kwale (N=13)	Mombasa (N=1)	Taita Taveta (N=18)	Total (N=32)
**Sex**				
Female	1.00 (7.7%)	0 (0%)	0 (0%)	1.00 (3.1%)
Male	12.0 (92.3%)	1.00 (100%)	17.0 (94.4%)	30.0 (93.8%)
Unknown	0 (0%)	0 (0%)	1.00 (5.6%)	1.00 (3.1%)
**Age**				
Mean (SD)	34.0 (11.9)	24.0 (NA)	37.3 (10.8)	35.5 (11.2)
Median [Min, Max]	31.0 [19.0, 56.0]	24.0 [24.0, 24.0]	35.5 [22.0, 54.0]	33.0 [19.0, 56.0]
**Symptoms**				
Asymptomatic	12.0 (92.3%)	1.00 (100%)	9.00 (50.0%)	22.0 (68.8%)
Unknown	1.00 (7.7%)	0 (0%)	8.00 (44.4%)	9.00 (28.1%)
Symptomatic	0 (0%)	0 (0%)	1.00 (5.6%)	1.00 (3.1%)
**Lineage**				
A	6.00 (46.2%)	0 (0%)	4.00 (22.2%)	10.0 (31.3%)
B.1	7.00 (53.8%)	0 (0%)	8.00 (44.4%)	15.0 (46.9%)
B.4	0 (0%)	1.00 (100%)	0 (0%)	1.00 (3.1%)
B.1.1.1	0 (0%)	0 (0%)	1.00 (5.6%)	1.00 (3.1%)
B.1.1.119	0 (0%)	0 (0%)	1.00 (5.6%)	1.00 (3.1%)
B.1.1.300	0 (0%)	0 (0%)	3.00 (16.7%)	3.00 (9.4%)
B.1.387	0 (0%)	0 (0%)	1.00 (5.6%)	1.00 (3.1%)

Supplementary table 4: An epidemiological summary of 32 of 67 border cases that had documented history of travel to Tanzania obtained at the Points of Entry (PoE) in Taita Taveta and Kwale counties.

Supplementary table 5

Name	Pool	Sequence	Length	tm (use 65)
**nCoV-2019_1_LEFT**	nCoV-2019_1	ACCAACCAACTTTCGATCTCTTGT	24	60.69
**nCoV-2019_1_RIGHT**	nCoV-2019_1	CATCTTTAAGATGTTGACGTGCCTC	25	60.45
**nCoV-2019_2_LEFT**	nCoV-2019_2	CTGTTTTACAGGTTCGCGACGT	22	61.67
**nCoV-2019_2_RIGHT**	nCoV-2019_2	TAAGGATCAGTGCCAAGCTCGT	22	61.74
**nCoV-2019_3_LEFT**	nCoV-2019_1	CGGTAATAAAGGAGCTGGTGGC	22	61.32
**nCoV-2019_3_RIGHT**	nCoV-2019_1	AAGGTGTCTGCAATTCATAGCTCT	24	60.32
**nCoV-2019_4_LEFT**	nCoV-2019_2	GGTGTATACTGCTGCCGTGAAC	22	61.56
**nCoV-2019_4_RIGHT**	nCoV-2019_2	CACAAGTAGTGGCACCTTCTTTAGT	25	60.97
**nCoV-2019_5_LEFT**	nCoV-2019_1	TGGTGAAACTTCATGGCAGACG	22	61.39
**nCoV-2019_5_RIGHT**	nCoV-2019_1	ATTGATGTTGACTTTCTCTTTTTGGAGT	28	60.17
**nCoV-2019_6_LEFT**	nCoV-2019_2	GGTGTTGTTGGAGAAGGTTCCG	22	61.64
**nCoV-2019_6_RIGHT**	nCoV-2019_2	TAGCGGCCTTCTGTAAAACACG	22	61.18
**nCoV-2019_7_LEFT**	nCoV-2019_1	ATCAGAGGCTGCTCGTGTTGTA	22	61.73
**nCoV-2019_7_LEFT_alt0**	nCoV-2019_1	CATTTGCATCAGAGGCTGCTCG	22	62.44
**nCoV-2019_7_RIGHT**	nCoV-2019_1	TGCACAGGTGACAATTTGTCCA	22	60.95
**nCoV-2019_7_RIGHT_alt5**	nCoV-2019_1	AGGTGACAATTTGTCCACCGAC	22	61.07
**nCoV-2019_8_LEFT**	nCoV-2019_2	AGAGTTTCTTAGAGACGGTTGGGA	24	61
**nCoV-2019_8_RIGHT**	nCoV-2019_2	GCTTCAACAGCTTCACTAGTAGGT	24	60.56
**nCoV-2019_9_LEFT**	nCoV-2019_1	TCCCACAGAAGTGTTAACAGAGGA	24	61.18
**nCoV-2019_9_LEFT_alt4**	nCoV-2019_1	TTCCCACAGAAGTGTTAACAGAGG	24	60.44
**nCoV-2019_9_RIGHT**	nCoV-2019_1	ATGACAGCATCTGCCACAACAC	22	61.71
**nCoV-2019_9_RIGHT_alt2**	nCoV-2019_1	GACAGCATCTGCCACAACACAG	22	62.26

**nCoV-2019_10_LEFT**	nCoV-2019_2	TGAGAAGTGCTCTGCCTATACAGT	24	61.12
**nCoV-2019_10_RIGHT**	nCoV-2019_2	TCATCTAACCAATCTTCTTCTTGCTCT	27	60.31
**nCoV-2019_11_LEFT**	nCoV-2019_1	GGAATTTGGTGCCACTTCTGCT	22	61.66
**nCoV-2019_11_RIGHT**	nCoV-2019_1	TCATCAGATTCAACTTGCATGGCA	24	61.35
**nCoV-2019_12_LEFT**	nCoV-2019_2	AAACATGGAGGAGGTGTTGCAG	22	61.08
**nCoV-2019_12_RIGHT**	nCoV-2019_2	TTCACTCTTCATTTCCAAAAAGCTTGA	27	60.36
**nCoV-2019_13_LEFT**	nCoV-2019_1	TCGCACAAATGTCTACTTAGCTGT	24	60.56
**nCoV-2019_13_RIGHT**	nCoV-2019_1	ACCACAGCAGTTAAAACACCCT	22	60.36
**nCoV-2019_14_LEFT**	nCoV-2019_2	CATCCAGATTCTGCCACTCTTGT	23	60.62
**nCoV-2019_14_LEFT_alt4**	nCoV-2019_2	TGGCAATCTTCATCCAGATTCTGC	24	61.47
**nCoV-2019_14_RIGHT**	nCoV-2019_2	AGTTTCCACACAGACAGGCATT	22	60.42
**nCoV-2019_14_RIGHT_alt2**	nCoV-2019_2	TGCGTGTTTCTTCTGCATGTGC	22	62.76
**nCoV-2019_15_LEFT**	nCoV-2019_1	ACAGTGCTTAAAAAGTGTAAAAGTGCC	27	61.32
**nCoV-2019_15_LEFT_alt1**	nCoV-2019_1	AGTGCTTAAAAAGTGTAAAAGTGCCT	26	60.13
**nCoV-2019_15_RIGHT**	nCoV-2019_1	AACAGAAACTGTAGCTGGCACT	22	60.16
**nCoV-2019_15_RIGHT_alt3**	nCoV-2019_1	ACTGTAGCTGGCACTTTGAGAGA	23	61.57
**nCoV-2019_16_LEFT**	nCoV-2019_2	AATTTGGAAGAAGCTGCTCGGT	22	60.82
**nCoV-2019_16_RIGHT**	nCoV-2019_2	CACAACTTGCGTGTGGAGGTTA	22	61.32
**nCoV-2019_17_LEFT**	nCoV-2019_1	CTTCTTTCTTTGAGAGAAGTGAGGACT	27	60.69
**nCoV-2019_17_RIGHT**	nCoV-2019_1	TTTGTTGGAGTGTTAACAATGCAGT	25	60.11
**nCoV-2019_18_LEFT**	nCoV-2019_2	TGGAAATACCCACAAGTTAATGGTTTAAC	29	60.69
**nCoV-2019_18_LEFT_alt2**	nCoV-2019_2	ACTTCTATTAAATGGGCAGATAACAACTGT	30	61.38
**nCoV-2019_18_RIGHT**	nCoV-2019_2	AGCTTGTTTACCACACGTACAAGG	24	61.51
**nCoV-2019_18_RIGHT_alt1**	nCoV-2019_2	GCTTGTTTACCACACGTACAAGG	23	60.3
**nCoV-2019_19_LEFT**	nCoV-2019_1	GCTGTTATGTACATGGGCACACT	23	61.18
**nCoV-2019_19_RIGHT**	nCoV-2019_1	TGTCCAACTTAGGGTCAATTTCTGT	25	60.4
**nCoV-2019_20_LEFT**	nCoV-2019_2	ACAAAGAAAACAGTTACACAACAACCA	27	60.68

**nCoV-2019_20_RIGHT**	nCoV-2019_2	ACGTGGCTTTATTAGTTGCATTGTT	25	60.28
**nCoV-2019_21_LEFT**	nCoV-2019_1	TGGCTATTGATTATAAACACTACACACCC	29	61.49
**nCoV-2019_21_LEFT_alt2**	nCoV-2019_1	GGCTATTGATTATAAACACTACACACCCT	29	61.29
**nCoV-2019_21_RIGHT**	nCoV-2019_1	TAGATCTGTGTGGCCAACCTCT	22	60.83
**nCoV-2019_21_RIGHT_alt0**	nCoV-2019_1	GATCTGTGTGGCCAACCTCTTC	22	61.2
**nCoV-2019_22_LEFT**	nCoV-2019_2	ACTACCGAAGTTGTAGGAGACATTATACT	29	61.25
**nCoV-2019_22_RIGHT**	nCoV-2019_2	ACAGTATTCTTTGCTATAGTAGTCGGC	27	60.73
**nCoV-2019_23_LEFT**	nCoV-2019_1	ACAACTACTAACATAGTTACACGGTGT	27	60.26
**nCoV-2019_23_RIGHT**	nCoV-2019_1	ACCAGTACAGTAGGTTGCAATAGTG	25	60.57
**nCoV-2019_24_LEFT**	nCoV-2019_2	AGGCATGCCTTCTTACTGTACTG	23	60.37
**nCoV-2019_24_RIGHT**	nCoV-2019_2	ACATTCTAACCATAGCTGAAATCGGG	26	61.19
**nCoV-2019_25_LEFT**	nCoV-2019_1	GCAATTGTTTTTCAGCTATTTTGCAGT	27	60.73
**nCoV-2019_25_RIGHT**	nCoV-2019_1	ACTGTAGTGACAAGTCTCTCGCA	23	61.3
**nCoV-2019_26_LEFT**	nCoV-2019_2	TTGTGATACATTCTGTGCTGGTAGT	25	60.28
**nCoV-2019_26_RIGHT**	nCoV-2019_2	TCCGCACTATCACCAACATCAG	22	60.42
**nCoV-2019_27_LEFT**	nCoV-2019_1	ACTACAGTCAGCTTATGTGTCAACC	25	60.8
**nCoV-2019_27_RIGHT**	nCoV-2019_1	AATACAAGCACCAAGGTCACGG	22	61.13
**nCoV-2019_28_LEFT**	nCoV-2019_2	ACATAGAAGTTACTGGCGATAGTTGT	26	60.13
**nCoV-2019_28_RIGHT**	nCoV-2019_2	TGTTTAGACATGACATGAACAGGTGT	26	60.91
**nCoV-2019_29_LEFT**	nCoV-2019_1	ACTTGTGTTCCTTTTTGTTGCTGC	24	61.39
**nCoV-2019_29_RIGHT**	nCoV-2019_1	AGTGTACTCTATAAGTTTTGATGGTGTGT	29	60.69
**nCoV-2019_30_LEFT**	nCoV-2019_2	GCACAACTAATGGTGACTTTTTGCA	25	61.19
**nCoV-2019_30_RIGHT**	nCoV-2019_2	ACCACTAGTAGATACACAAACACCAG	26	60.3
**nCoV-2019_31_LEFT**	nCoV-2019_1	TTCTGAGTACTGTAGGCACGGC	22	62.03
**nCoV-2019_31_RIGHT**	nCoV-2019_1	ACAGAATAAACACCAGGTAAGAATGAGT	28	60.69
**nCoV-2019_32_LEFT**	nCoV-2019_2	TGGTGAATACAGTCATGTAGTTGCC	25	61.09
**nCoV-2019_32_RIGHT**	nCoV-2019_2	AGCACATCACTACGCAACTTTAGA	24	60.56

**nCoV-2019_33_LEFT**	nCoV-2019_1	ACTTTTGAAGAAGCTGCGCTGT	22	61.58
**nCoV-2019_33_RIGHT**	nCoV-2019_1	TGGACAGTAAACTACGTCATCAAGC	25	61.08
**nCoV-2019_34_LEFT**	nCoV-2019_2	TCCCATCTGGTAAAGTTGAGGGT	23	61.02
**nCoV-2019_34_RIGHT**	nCoV-2019_2	AGTGAAATTGGGCCTCATAGCA	22	60.03
**nCoV-2019_35_LEFT**	nCoV-2019_1	TGTTCGCATTCAACCAGGACAG	22	61.39
**nCoV-2019_35_RIGHT**	nCoV-2019_1	ACTTCATAGCCACAAGGTTAAAGTCA	26	60.69
**nCoV-2019_36_LEFT**	nCoV-2019_2	TTAGCTTGGTTGTACGCTGCTG	22	61.44
**nCoV-2019_36_RIGHT**	nCoV-2019_2	GAACAAAGACCATTGAGTACTCTGGA	26	60.74
**nCoV-2019_37_LEFT**	nCoV-2019_1	ACACACCACTGGTTGTTACTCAC	23	60.93
**nCoV-2019_37_RIGHT**	nCoV-2019_1	GTCCACACTCTCCTAGCACCAT	22	61.48
**nCoV-2019_38_LEFT**	nCoV-2019_2	ACTGTGTTATGTATGCATCAGCTGT	25	60.86
**nCoV-2019_38_RIGHT**	nCoV-2019_2	CACCAAGAGTCAGTCTAAAGTAGCG	25	61.13
**nCoV-2019_39_LEFT**	nCoV-2019_1	AGTATTGCCCTATTTTCTTCATAACTGGT	29	61
**nCoV-2019_39_RIGHT**	nCoV-2019_1	TGTAACTGGACACATTGAGCCC	22	60.55
**nCoV-2019_40_LEFT**	nCoV-2019_2	TGCACATCAGTAGTCTTACTCTCAGT	26	61.25
**nCoV-2019_40_RIGHT**	nCoV-2019_2	CATGGCTGCATCACGGTCAAAT	22	62.09
**nCoV-2019_41_LEFT**	nCoV-2019_1	GTTCCCTTCCATCATATGCAGCT	23	60.75
**nCoV-2019_41_RIGHT**	nCoV-2019_1	TGGTATGACAACCATTAGTTTGGCT	25	60.75
**nCoV-2019_42_LEFT**	nCoV-2019_2	TGCAAGAGATGGTTGTGTTCCC	22	61.08
**nCoV-2019_42_RIGHT**	nCoV-2019_2	CCTACCTCCCTTTGTTGTGTTGT	23	60.69
**nCoV-2019_43_LEFT**	nCoV-2019_1	TACGACAGATGTCTTGTGCTGC	22	60.93
**nCoV-2019_43_RIGHT**	nCoV-2019_1	AGCAGCATCTACAGCAAAAGCA	22	61.14
**nCoV-2019_44_LEFT**	nCoV-2019_2	TGCCACAGTACGTCTACAAGCT	22	61.66
**nCoV-2019_44_LEFT_alt3**	nCoV-2019_2	CCACAGTACGTCTACAAGCTGG	22	60.67
**nCoV-2019_44_RIGHT**	nCoV-2019_2	AACCTTTCCACATACCGCAGAC	22	60.87
**nCoV-2019_44_RIGHT_alt0**	nCoV-2019_2	CGCAGACGGTACAGACTGTGTT	22	62.77
**nCoV-2019_45_LEFT**	nCoV-2019_1	TACCTACAACTTGTGCTAATGACCC	25	60.57

**nCoV-2019_45_LEFT_alt2**	nCoV-2019_1	AGTATGTACAAATACCTACAACTTGTGCT	29	60.94
**nCoV-2019_45_RIGHT**	nCoV-2019_1	AAATTGTTTCTTCATGTTGGTAGTTAGAGA	30	60.01
**nCoV-2019_45_RIGHT_alt7**	nCoV-2019_1	TTCATGTTGGTAGTTAGAGAAAGTGTGTC	29	61.53
**nCoV-2019_46_LEFT**	nCoV-2019_2	TGTCGCTTCCAAGAAAAGGACG	22	61.38
**nCoV-2019_46_LEFT_alt1**	nCoV-2019_2	CGCTTCCAAGAAAAGGACGAAGA	23	61.35
**nCoV-2019_46_RIGHT**	nCoV-2019_2	CACGTTCACCTAAGTTGGCGTA	22	60.86
**nCoV-2019_46_RIGHT_alt2**	nCoV-2019_2	CACGTTCACCTAAGTTGGCGTAT	23	61.17
**nCoV-2019_47_LEFT**	nCoV-2019_1	AGGACTGGTATGATTTTGTAGAAAACCC	28	61.42
**nCoV-2019_47_RIGHT**	nCoV-2019_1	AATAACGGTCAAAGAGTTTTAACCTCTC	28	60.06
**nCoV-2019_48_LEFT**	nCoV-2019_2	TGTTGACACTGACTTAACAAAGCCT	25	61.09
**nCoV-2019_48_RIGHT**	nCoV-2019_2	TAGATTACCAGAAGCAGCGTGC	22	60.74
**nCoV-2019_49_LEFT**	nCoV-2019_1	AGGAATTACTTGTGTATGCTGCTGA	25	60.57
**nCoV-2019_49_RIGHT**	nCoV-2019_1	TGACGATGACTTGGTTAGCATTAATACA	28	61.05
**nCoV-2019_50_LEFT**	nCoV-2019_2	GTTGATAAGTACTTTGATTGTTACGATGGT	30	60.59
**nCoV-2019_50_RIGHT**	nCoV-2019_2	TAACATGTTGTGCCAACCACCA	22	60.95
**nCoV-2019_51_LEFT**	nCoV-2019_1	TCAATAGCCGCCACTAGAGGAG	22	61.34
**nCoV-2019_51_RIGHT**	nCoV-2019_1	AGTGCATTAACATTGGCCGTGA	22	61.14
**nCoV-2019_52_LEFT**	nCoV-2019_2	CATCAGGAGATGCCACAACTGC	22	61.83
**nCoV-2019_52_RIGHT**	nCoV-2019_2	GTTGAGAGCAAAATTCATGAGGTCC	25	60.62
**nCoV-2019_53_LEFT**	nCoV-2019_1	AGCAAAATGTTGGACTGAGACTGA	24	60.69
**nCoV-2019_53_RIGHT**	nCoV-2019_1	AGCCTCATAAAACTCAGGTTCCC	23	60.31
**nCoV-2019_54_LEFT**	nCoV-2019_2	TGAGTTAACAGGACACATGTTAGACA	26	60.18
**nCoV-2019_54_RIGHT**	nCoV-2019_2	AACCAAAAACTTGTCCATTAGCACA	25	60.11
**nCoV-2019_55_LEFT**	nCoV-2019_1	ACTCAACTTTACTTAGGAGGTATGAGCT	28	61.43
**nCoV-2019_55_RIGHT**	nCoV-2019_1	GGTGTACTCTCCTATTTGTACTTTACTGT	29	60.54
**nCoV-2019_56_LEFT**	nCoV-2019_2	ACCTAGACCACCACTTAACCGA	22	60.49
**nCoV-2019_56_RIGHT**	nCoV-2019_2	ACACTATGCGAGCAGAAGGGTA	22	61.21

**nCoV-2019_57_LEFT**	nCoV-2019_1	ATTCTACACTCCAGGGACCACC	22	61.16
**nCoV-2019_57_RIGHT**	nCoV-2019_1	GTAATTGAGCAGGGTCGCCAAT	22	61.26
**nCoV-2019_58_LEFT**	nCoV-2019_2	TGATTTGAGTGTTGTCAATGCCAGA	25	61.44
**nCoV-2019_58_RIGHT**	nCoV-2019_2	CTTTTCTCCAAGCAGGGTTACGT	23	61.06
**nCoV-2019_59_LEFT**	nCoV-2019_1	TCACGCATGATGTTTCATCTGCA	23	61.42
**nCoV-2019_59_RIGHT**	nCoV-2019_1	AAGAGTCCTGTTACATTTTCAGCTTG	26	60.02
**nCoV-2019_60_LEFT**	nCoV-2019_2	TGATAGAGACCTTTATGACAAGTTGCA	27	60.53
**nCoV-2019_60_RIGHT**	nCoV-2019_2	GGTACCAACAGCTTCTCTAGTAGC	24	60.44
**nCoV-2019_61_LEFT**	nCoV-2019_1	TGTTTATCACCCGCGAAGAAGC	22	61.5
**nCoV-2019_61_RIGHT**	nCoV-2019_1	ATCACATAGACAACAGGTGCGC	22	61.25
**nCoV-2019_62_LEFT**	nCoV-2019_2	GGCACATGGCTTTGAGTTGACA	22	61.91
**nCoV-2019_62_RIGHT**	nCoV-2019_2	GTTGAACCTTTCTACAAGCCGC	22	60.35
**nCoV-2019_63_LEFT**	nCoV-2019_1	TGTTAAGCGTGTTGACTGGACT	22	60.16
**nCoV-2019_63_RIGHT**	nCoV-2019_1	ACAAACTGCCACCATCACAACC	22	61.85
**nCoV-2019_64_LEFT**	nCoV-2019_2	TCGATAGATATCCTGCTAATTCCATTGT	28	60.11
**nCoV-2019_64_RIGHT**	nCoV-2019_2	AGTCTTGTAAAAGTGTTCCAGAGGT	25	60.1
**nCoV-2019_65_LEFT**	nCoV-2019_1	GCTGGCTTTAGCTTGTGGGTTT	22	61.92
**nCoV-2019_65_RIGHT**	nCoV-2019_1	TGTCAGTCATAGAACAAACACCAATAGT	28	60.9
**nCoV-2019_66_LEFT**	nCoV-2019_2	GGGTGTGGACATTGCTGCTAAT	22	61.21
**nCoV-2019_66_RIGHT**	nCoV-2019_2	TCAATTTCCATTTGACTCCTGGGT	24	60.45
**nCoV-2019_67_LEFT**	nCoV-2019_1	GTTGTCCAACAATTACCTGAAACTTACT	28	60.43
**nCoV-2019_67_RIGHT**	nCoV-2019_1	CAACCTTAGAAACTACAGATAAATCTTGGG	30	60.4
**nCoV-2019_68_LEFT**	nCoV-2019_2	ACAGGTTCATCTAAGTGTGTGTGT	24	60.14
**nCoV-2019_68_RIGHT**	nCoV-2019_2	CTCCTTTATCAGAACCAGCACCA	23	60.31
**nCoV-2019_69_LEFT**	nCoV-2019_1	TGTCGCAAAATATACTCAACTGTGTCA	27	61.43
**nCoV-2019_69_RIGHT**	nCoV-2019_1	TCTTTATAGCCACGGAACCTCCA	23	61.14
**nCoV-2019_70_LEFT**	nCoV-2019_2	ACAAAAGAAAATGACTCTAAAGAGGGTTT	29	60.13

**nCoV-2019_70_RIGHT**	nCoV-2019_2	TGACCTTCTTTTAAAGACATAACAGCAG	28	60.27
**nCoV-2019_71_LEFT**	nCoV-2019_1	ACAAATCCAATTCAGTTGTCTTCCTATTC	29	60.54
**nCoV-2019_71_RIGHT**	nCoV-2019_1	TGGAAAAGAAAGGTAAGAACAAGTCCT	27	60.8
**nCoV-2019_72_LEFT**	nCoV-2019_2	ACACGTGGTGTTTATTACCCTGAC	24	61.04
**nCoV-2019_72_RIGHT**	nCoV-2019_2	ACTCTGAACTCACTTTCCATCCAAC	25	60.97
**nCoV-2019_73_LEFT**	nCoV-2019_1	CAATTTTGTAATGATCCATTTTTGGGTGT	29	60.29
**nCoV-2019_73_RIGHT**	nCoV-2019_1	CACCAGCTGTCCAACCTGAAGA	22	62.45
**nCoV-2019_74_LEFT**	nCoV-2019_2	ACATCACTAGGTTTCAAACTTTACTTGC	28	60.68
**nCoV-2019_74_RIGHT**	nCoV-2019_2	GCAACACAGTTGCTGATTCTCTTC	24	60.85
**nCoV-2019_75_LEFT**	nCoV-2019_1	AGAGTCCAACCAACAGAATCTATTGT	26	60.24
**nCoV-2019_75_RIGHT**	nCoV-2019_1	ACCACCAACCTTAGAATCAAGATTGT	26	60.69
**nCoV-2019_76_LEFT**	nCoV-2019_2	AGGGCAAACTGGAAAGATTGCT	22	60.76
**nCoV-2019_76_LEFT_alt3**	nCoV-2019_2	GGGCAAACTGGAAAGATTGCTGA	23	61.87
**nCoV-2019_76_RIGHT**	nCoV-2019_2	ACACCTGTGCCTGTTAAACCAT	22	60.42
**nCoV-2019_76_RIGHT_alt0**	nCoV-2019_2	ACCTGTGCCTGTTAAACCATTGA	23	60.69
**nCoV-2019_77_LEFT**	nCoV-2019_1	CCAGCAACTGTTTGTGGACCTA	22	60.75
**nCoV-2019_77_RIGHT**	nCoV-2019_1	CAGCCCCTATTAAACAGCCTGC	22	61.59
**nCoV-2019_78_LEFT**	nCoV-2019_2	CAACTTACTCCTACTTGGCGTGT	23	60.55
**nCoV-2019_78_RIGHT**	nCoV-2019_2	TGTGTACAAAAACTGCCATATTGCA	25	60.22
**nCoV-2019_79_LEFT**	nCoV-2019_1	GTGGTGATTCAACTGAATGCAGC	23	60.92
**nCoV-2019_79_RIGHT**	nCoV-2019_1	CATTTCATCTGTGAGCAAAGGTGG	24	60.62
**nCoV-2019_80_LEFT**	nCoV-2019_2	TTGCCTTGGTGATATTGCTGCT	22	60.89
**nCoV-2019_80_RIGHT**	nCoV-2019_2	TGGAGCTAAGTTGTTTAACAAGCG	24	60.02
**nCoV-2019_81_LEFT**	nCoV-2019_1	GCACTTGGAAAACTTCAAGATGTGG	25	61.24
**nCoV-2019_81_RIGHT**	nCoV-2019_1	GTGAAGTTCTTTTCTTGTGCAGGG	24	60.73
**nCoV-2019_82_LEFT**	nCoV-2019_2	GGGCTATCATCTTATGTCCTTCCCT	25	61.52
**nCoV-2019_82_RIGHT**	nCoV-2019_2	TGCCAGAGATGTCACCTAAATCAA	24	60.02

**nCoV-2019_83_LEFT**	nCoV-2019_1	TCCTTTGCAACCTGAATTAGACTCA	25	60.46
**nCoV-2019_83_RIGHT**	nCoV-2019_1	TTTGACTCCTTTGAGCACTGGC	22	61.33
**nCoV-2019_84_LEFT**	nCoV-2019_2	TGCTGTAGTTGTCTCAAGGGCT	22	61.61
**nCoV-2019_84_RIGHT**	nCoV-2019_2	AGGTGTGAGTAAACTGTTACAAACAAC	27	60.36
**nCoV-2019_85_LEFT**	nCoV-2019_1	ACTAGCACTCTCCAAGGGTGTT	22	61.03
**nCoV-2019_85_RIGHT**	nCoV-2019_1	ACACAGTCTTTTACTCCAGATTCCC	25	60.51
**nCoV-2019_86_LEFT**	nCoV-2019_2	TCAGGTGATGGCACAACAAGTC	22	61.07
**nCoV-2019_86_RIGHT**	nCoV-2019_2	ACGAAAGCAAGAAAAAGAAGTACGC	25	61.01
**nCoV-2019_87_LEFT**	nCoV-2019_1	CGACTACTAGCGTGCCTTTGTA	22	60.16
**nCoV-2019_87_RIGHT**	nCoV-2019_1	ACTAGGTTCCATTGTTCAAGGAGC	24	60.81
**nCoV-2019_88_LEFT**	nCoV-2019_2	CCATGGCAGATTCCAACGGTAC	22	61.58
**nCoV-2019_88_RIGHT**	nCoV-2019_2	TGGTCAGAATAGTGCCATGGAGT	23	61.4
**nCoV-2019_89_LEFT**	nCoV-2019_1	GTACGCGTTCCATGTGGTCATT	22	61.5
**nCoV-2019_89_LEFT_alt2**	nCoV-2019_1	CGCGTTCCATGTGGTCATTCAA	22	62.01
**nCoV-2019_89_RIGHT**	nCoV-2019_1	ACCTGAAAGTCAACGAGATGAAACA	25	60.91
**nCoV-2019_89_RIGHT_alt4**	nCoV-2019_1	ACGAGATGAAACATCTGTTGTCACT	25	60.74
**nCoV-2019_90_LEFT**	nCoV-2019_2	ACACAGACCATTCCAGTAGCAGT	23	61.58
**nCoV-2019_90_RIGHT**	nCoV-2019_2	TGAAATGGTGAATTGCCCTCGT	22	60.82
**nCoV-2019_91_LEFT**	nCoV-2019_1	TCACTACCAAGAGTGTGTTAGAGGT	25	60.93
**nCoV-2019_91_RIGHT**	nCoV-2019_1	TTCAAGTGAGAACCAAAAGATAATAAGCA	29	60.03
**nCoV-2019_92_LEFT**	nCoV-2019_2	TTTGTGCTTTTTAGCCTTTCTGCT	24	60.14
**nCoV-2019_92_RIGHT**	nCoV-2019_2	AGGTTCCTGGCAATTAATTGTAAAAGG	27	60.53
**nCoV-2019_93_LEFT**	nCoV-2019_1	TGAGGCTGGTTCTAAATCACCCA	23	61.59
**nCoV-2019_93_RIGHT**	nCoV-2019_1	AGGTCTTCCTTGCCATGTTGAG	22	60.55
**nCoV-2019_94_LEFT**	nCoV-2019_2	GGCCCCAAGGTTTACCCAATAA	22	60.56
**nCoV-2019_94_RIGHT**	nCoV-2019_2	TTTGGCAATGTTGTTCCTTGAGG	23	60.18
**nCoV-2019_95_LEFT**	nCoV-2019_1	TGAGGGAGCCTTGAATACACCA	22	61.1

**nCoV-2019_95_RIGHT**	nCoV-2019_1	CAGTACGTTTTTGCCGAGGCTT	22	61.95
**nCoV-2019_96_LEFT**	nCoV-2019_2	GCCAACAACAACAAGGCCAAAC	22	61.82
**nCoV-2019_96_RIGHT**	nCoV-2019_2	TAGGCTCTGTTGGTGGGAATGT	22	61.36
**nCoV-2019_97_LEFT**	nCoV-2019_1	TGGATGACAAAGATCCAAATTTCAAAGA	28	60.22
**nCoV-2019_97_RIGHT**	nCoV-2019_1	ACACACTGATTAAAGATTGCTATGTGAG	28	60.17
**nCoV-2019_98_LEFT**	nCoV-2019_2	AACAATTGCAACAATCCATGAGCA	24	60.5
**nCoV-2019_98_RIGHT**	nCoV-2019_2	TTCTCCTAAGAAGCTATTAAAATCACATGG	30	60.01

Supplementary table 5: List of ARTIC primers used in the amplification of SARS-CoV-2 genomes as published by Quick et al (https://raw.githubusercontent.com/artic-network/artic-ncov2019/master/primer_schemes/nCoV-2019/V3/nCoV-2019.tsv)
