# Supplementary material for: Tracking the introduction and spread of SARS-CoV-2 in coastal Kenya
Source: Nat Commun. 2021 Aug 10;12:4809. doi: 10.1038/s41467-021-25137-x (PMC8355311; doi:10.1038/s41467-021-25137-x)
Supplement: Supplementary file 3 — Reporting Summary [file 41467_2021_25137_MOESM3_ESM.pdf]

nature portfolio

Corresponding author(s): George Githinji

Last updated by author(s): Jul 26, 2021

Reporting Summary

Nature Portfolio wishes to improve the reproducibility of the work that we publish. This form provides structure for consistency and transparency in reporting. For further information on Nature Portfolio policies, see our Editorial Policies and the Editorial Policy Checklist.

Statistics

For all statistical analyses, confirm that the following items are present in the figure legend, table legend, main text, or Methods section.

n/a	Confirmed	
☐	☒	The exact sample size (*n*) for each experimental group/condition, given as a discrete number and unit of measurement
☐	☒	A statement on whether measurements were taken from distinct samples or whether the same sample was measured repeatedly
☐	☒	The statistical test(s) used AND whether they are one- or two-sided<br>*Only common tests should be described solely by name; describe more complex techniques in the Methods section.*
☐	☒	A description of all covariates tested
☐	☒	A description of any assumptions or corrections, such as tests of normality and adjustment for multiple comparisons
☐	☒	A full description of the statistical parameters including central tendency (e.g. means) or other basic estimates (e.g. regression coefficient) AND variation (e.g. standard deviation) or associated estimates of uncertainty (e.g. confidence intervals)
☒	☐	For null hypothesis testing, the test statistic (e.g. *F*, *t*, *r*) with confidence intervals, effect sizes, degrees of freedom and *P* value noted<br>*Give P values as exact values whenever suitable.*
☒	☐	For Bayesian analysis, information on the choice of priors and Markov chain Monte Carlo settings
☒	☐	For hierarchical and complex designs, identification of the appropriate level for tests and full reporting of outcomes
☐	☐	Estimates of effect sizes (e.g. Cohen's *d*, Pearson's *r*), indicating how they were calculated

Our web collection on statistics for biologists contains articles on many of the points above.

Software and code

Policy information about availability of computer code

Data collection	The raw data were collected by the rapid response teams. These samples were processed and sequenced using the oxford nanopore technology. Raw sequence data were base-called using the proprietary "Guppy version 4" software series. Resulting fasta and fastq files were used in the analysis.
Data analysis	We used R version 4.0.2 for statistical analysis. Quality control and clade assignment of the sequence data was conducted using NextClade version 0.13.0. Data processing was conducted using the Augur version 11 tool. Multiple sequence alignments were conducted using MAFFT version 7.475. Pango Lineage were assigned using Pangolin toolkit version 2.3.2 with pangoLEARN version 2021-02-10. Phylogenetic trees were estimated using IQTree version 2.0.3. Ancestral reconstruction was conducted using TreeTime version 0.81 and pastML version 1.9.15. The full source code used in the analysis work and workflows are available at https://github.com/george-githinji/sars-cov-2-early-phase-manuscript .

For manuscripts utilizing custom algorithms or software that are central to the research but not yet described in published literature, software must be made available to editors and reviewers. We strongly encourage code deposition in a community repository (e.g. GitHub). See the Nature Portfolio guidelines for submitting code & software for further information.

Data

Policy information about availability of data

All manuscripts must include a data availability statement. This statement should provide the following information, where applicable:

- Accession codes, unique identifiers, or web links for publicly available datasets
- A description of any restrictions on data availability
- For clinical datasets or third party data, please ensure that the statement adheres to our policy

This study did not generate unique reagents, but raw data and code generated as part of this research can be found in the Supplemental files as well as on public resources as specified in the Data and Code Availability section below. SARS-CoV-2 sequence data used in this analysis are publicly available from GenBank accession numbers (MW751078 - MW751422, MW931663-MW931714). A summary json file for interactive phylogenetic analysis is available as Supplementary Data 1 and can be loaded and visualised using Auspice web-based tool. Additional files and source code are available from GitHub https://github.com/george-githinji/sars-cov-2-early-phase-manuscript/data.

Field-specific reporting

Please select the one below that is the best fit for your research. If you are not sure, read the appropriate sections before making your selection.

[x] Life sciences [ ] Behavioural & social sciences [ ] Ecological, evolutionary & environmental sciences

For a reference copy of the document with all sections, see nature.com/documents/nr-reporting-summary-flat.pdf

Life sciences study design

All studies must disclose on these points even when the disclosure is negative.

Sample size	This is a retrospective genomic epidemiology study of SARS-CoV-2 PCR Positive cases in Kenya in the early phase of the epidemic. The data was collected as part of the epidemic response by the Ministry of Health. We sequences all available samples initially and later all samples with a Ct score <30. No sample size was calculated and we made use of the available sequence data after quality control and as described in the methods section of the paper.
Data exclusions	Sample<br><br>Sequence data were excluded if they did not meet 80% genome coverage. Samples for sequencing were excluded if the Ct score was >30. public data from GISAID were subsampled based on a criteria outlined in the methods section. Data with incomplete dates were excluded from analysis.
Replication	This was sequence analysis project. We used a reproducible workflow to conduct the analysis. The workflow was run at least two times after any modification to confirm that the data were reproducible.
Randomization	This was not applicable to our study because we used whole genome sequence data that were analysed retrospectively. We included each and every data point that passed the inclusion criteria and such no randomization was conducted.
Blinding	Blinding was not applicable to the study. This was an retrospective genomic epidemiological study. Care was taken to ensure personal or identifying information was not provided.

Reporting for specific materials, systems and methods

We require information from authors about some types of materials, experimental systems and methods used in many studies. Here, indicate whether each material, system or method listed is relevant to your study. If you are not sure if a list item applies to your research, read the appropriate section before selecting a response.

Materials & experimental systems

n/a	Involved in the study	
[x]	[ ]	Antibodies
[x]	[ ]	Eukaryotic cell lines
[x]	[ ]	Palaeontology and archaeology
[x]	[ ]	Animals and other organisms
[ ]	[x]	Human research participants
[x]	[ ]	Clinical data
[x]	[ ]	Dual use research of concern

Methods

n/a	Involved in the study	
[x]	[ ]	ChIP-seq
[x]	[ ]	Flow cytometry
[x]	[ ]	MRI-based neuroimaging

Human research participants

Policy information about studies involving human research participants

Population characteristics	Nasal-pharyngeal samples were collected from individuals that presented for SARS-CoV-2 diagnostic testing as part of the Ministry of Health public health action. Where possible the following data were obtained, age, gender/sex, location and clinical symptoms at the time of sampling. Only virus pathogen sequence data was obtained using a specific amplicon based amplification of SARS-CoV-2 viral material.
Recruitment	The study utilized data that was obtained as part of a public health epidemic response. As such no specific recruitment criteria was followed. We analyzed SARS-CoV-2 specific virus sequence data from samples collected retrospectively. A sample from a diagnostic test with relatively high viral load (Ct < 30) and with a genome coverage of at 80% was used in the analysis.
Ethics oversight	The whole genome sequencing study protocol was reviewed and approved by the Scientific and Ethics Review Committee (SERU) residing at the Kenya Medical Research Institute (KEMRI) headquarters in Nairobi (SERU # 4035). Ethical approval of this study was provided by SERU.

Note that full information on the approval of the study protocol must also be provided in the manuscript.
