# Supplementary material for: Tracking the introduction and spread of SARS-CoV-2 in coastal Kenya
Source: Nat Commun. 2021 Aug 10;12:4809. doi: 10.1038/s41467-021-25137-x (PMC8355311; doi:10.1038/s41467-021-25137-x)
Supplement: Supplementary file 4 — Description of Additional Supplementary Files [file 41467_2021_25137_MOESM4_ESM.pdf]

Description of Additional Supplementary Files

File Name: Supplementary Data 1
Description: A NextStrain based Json file generated using the Augur pipeline and containing an interactive analysis of the Coastal Kenyan Sequences within the global context of circulating SARS-CoV-2 sequences between March and July 2020.

File Name: Supplementary Data 2
Description: A list of global GISAID SARS-CoV-2 sequences that were sampled used to provide a global context for coastal Kenya SARS-CoV-2 sequences.
